# Practising psychiatry in Sri Lanka: challenges and opportunities

**DOI:** 10.1192/bji.2022.28

**Published:** 2023-02

**Authors:** David Skuse

**Affiliations:** Professor of Behavioural and Brain Sciences, Division of Population, Policy and Practice, UCL Great Ormond Street Institute of Child Health, London, UK. Email d.skuse@ucl.ac.uk

**Keywords:** Sri Lanka, education and training, prescription drug misuse, mental health legislation, involuntary psychiatric treatment

## Abstract

This month's issue of *BJPsych International* focuses on psychiatry in Sri Lanka, with articles on suggested improvements in education and training, the country's outdated legislation regarding involuntary psychiatric treatment, and the misuse of prescription medications.

Our theme this month concerns the practice of psychiatry in Sri Lanka, with three related articles by Hapangama and colleagues. The first of these concerns the training of medical students and postgraduate education in psychiatry.^1^ Throughout the world, as medicine advances, one might expect the attitude of medical students to have evolved towards greater interest in and empathy for mental illness, among their peers and patients. Recent research (e.g. Wang et al, 2016;^2^ Pascucci et al, 2017^3^) suggests that negative attitudes persist. In Sri Lanka, the place of psychiatry in the medical school curriculum is not prominent, and the authors propose introducing a postgraduate internship in psychiatry as an option during foundation year training. It would be interesting to see how enthusiastically this option is selected.

A second article considers the need for an updated version of Sri Lanka's mental health legislation concerning the management of involuntary patients.^4^ Some elements of the existing law date back to the days of British governance in the 19th century, and reform has been pending for decades. It is concerning to learn that current official guidance mandates involuntary treatment in just one hospital, which is located in Colombo, so patients may be held ‘on remand’ for weeks in other parts of the country before their transfer can be arranged.

Finally, the authors draw our attention to the problem of drug misuse in Sri Lanka, with a focus on the ready availability of prescription drugs that are effectively unregulated.^5^ As we know from the epidemic of misuse of drugs such as OxyContin in the USA and Europe, even if there are controls on who has a license to prescribe, these can easily be undermined. The concerns expressed here about the ready availability of mind-altering legal medications are not confined to Sri Lanka.^6^
